# Real-time large-area imaging of the corneal subbasal nerve plexus

**DOI:** 10.1038/s41598-022-05983-5

**Published:** 2022-02-15

**Authors:** Stephan Allgeier, Andreas Bartschat, Sebastian Bohn, Rudolf F. Guthoff, Veit Hagenmeyer, Lukas Kornelius, Ralf Mikut, Klaus-Martin Reichert, Karsten Sperlich, Nadine Stache, Oliver Stachs, Bernd Köhler

**Affiliations:** 1 Institute for Automation and Applied Informatics, Karlsruhe Institute of Technology (KIT), Karlsruhe, Germany; 2 Department of Ophthalmology, Rostock University Medical Center, Rostock, Germany; 3 Department Life, Light and Matter, University of Rostock, Rostock, Germany; 4 Department of Obstetrics and Gynecology, University of Rostock, Rostock, Germany

**Keywords:** Biomedical engineering, Biomarkers

## Abstract

The morphometric assessment of the corneal subbasal nerve plexus (SNP) by confocal microscopy holds great potential as a sensitive biomarker for various ocular and systemic conditions and diseases. Automated wide-field montages (or large-area mosaic images) of the SNP provide an opportunity to overcome the limited field of view of the available imaging systems without the need for manual, subjective image selection for morphometric characterization. However, current wide-field montaging solutions usually calculate the mosaic image after the examination session, without a reliable means for the clinician to predict or estimate the resulting mosaic image quality during the examination. This contribution describes a novel approach for a real-time creation and visualization of a mosaic image of the SNP that facilitates an informed evaluation of the quality of the acquired image data immediately at the time of recording. In cases of insufficient data quality, the examination can be aborted and repeated immediately, while the patient is still at the microscope. Online mosaicking also offers the chance to identify an overlap of the imaged tissue region with previous SNP mosaic images, which can be particularly advantageous for follow-up examinations.

## Introduction

The transparent and densely innervated human cornea is the only tissue of the human body in which nerve fibers are non-invasively accessible to optical in vivo imaging. With its lateral resolution, in vivo corneal confocal microscopy (CCM) is the current state of the art technique for obtaining high-quality images of the subbasal nerve plexus (SNP) at the epithelial basement membrane^1,2^. Using this technology, the morphology of the SNP has been studied as a conveniently accessible biomarker in various conditions such as dry eye disease^3^, diabetic neuropathy^4,5^, chemotherapy^6^, HIV infection^7^, and Parkinson’s disease^8^. However, the inhomogeneity of the local distribution of SNP nerve structures limits the sensitivity and specificity of this method, particularly regarding the relatively small field of view of the available instruments in the order of only 0.10–0.16 mm^2^. It is commonly accepted that reliable quantitative characterization of the SNP morphology needs to be based on a significantly larger area of the SNP^9–12^. The most common way to encounter this constraint is to obtain and analyze several (ideally non-overlapping) CCM images. Sample sizes between three and eight single images are often reported in literature^1^. The image acquisition process—and frequently the subsequent analysis of the image data as well—is currently characterized almost exclusively by manual tasks and subjective decisions. Clearly defined procedures^13^ help reduce subjective bias effects^14^ but cannot eliminate them.

Automated methods for creating wide-field montages or mosaic images of the subbasal nerve structures from acquired image sequences have been proposed as an alternative approach for morphological characterization of the SNP over an expanded area^15–20^. Because of the automated processes, these approaches are inherently unaffected by subjective selection bias. As an additional potential benefit, the resulting mosaic images establish a better localization of the imaged corneal region^21^ and they may reveal phenomena that are not discernible in the field of view of single CCM images^22^. However, the successful generation of high-quality SNP mappings is challenging, particularly with respect to the image acquisition task. Typical dataset sizes for wide-field montages are in the order of several hundred images^16–18,22^, so manually controlled data collection methods are not feasible as a routine task. This has been solved by different approaches to guide the patients’ view direction, and therefore their eye movements, while image sequences are being recorded^16,17,19^. These techniques provide a fast and convenient way to expand the imaged SNP area, exploiting the fact that the SNP is located at the epithelial basement membrane, primarily parallel to the corneal surface. Because of the narrow depth of field of the CCM technology, however, effects such as axial eye movements, varying epithelial thickness, the curvature of the cornea, or small deviations from the surface-parallel arrangement of the SNP negatively affect the montaging result. The affected mosaic image regions often exhibit low contrast or even discontinuous representation of the nerve structures^19,22^. This issue has been addressed by a continuous computer-controlled oscillation of the focus plane centered on the average SNP level^20^. In effect, this technique implements a 3D confocal imaging protocol with a limited axial extent that is just deep enough to guarantee that the full extent of the axial deviations of the SNP layer is included.

All of the SNP mapping methods mentioned above possess a common restriction: The image acquisition and the mosaic image creation are implemented as two separate processes that have to be executed in a strictly consecutive manner. Consequently, the live presentation of the fast-changing sequence of acquired image frames is the only source of information available during the recording process, which is insufficient in practice to reliably predict the size or quality of the SNP mosaic image that is calculated from the dataset subsequently. The real-time mapping technique proposed by Zhivov et al. in 2010 addresses this deficit^15^. However, while the large-area image map is created in real-time by software, the lateral expansion of the acquired area has to be performed by careful manual interaction and requires a high level of experience. Experiments of our group to use this real-time mapping technique in combination with automatically guided eye movements were not successful. Furthermore, it suffers from the same issues as other 2D-only imaging approaches as described above.

The present contribution introduces a novel approach for automated imaging and real-time visualization of a continuously expanding region of the SNP. The system builds on and incorporates previous developments for guided eye movement and computer-controlled focus oscillation. These are combined with novel algorithms for real-time image registration and mosaic image generation. The primary aim of the presented method is to facilitate an informed evaluation of the quality of the acquired image data immediately at the time of recording. The potential and limitations of the proposed system are evaluated and discussed based on experimental results.

## Methods

The system used in the present paper employs the highly automated imaging process that has been described in detail previously in combination with an independent, subsequent offline SNP mosaicking step^20^. We will therefore only provide a brief general outline of the imaging process here, whereas the novel real-time SNP mosaicking will be described in more detail. Note that the raw CCM image sequence is still saved and can be used for further purposes such as offline mosaicking without real-time constraints. The imaging process employs two dedicated hardware components, the RCM 2.0^23^ and the EyeGuidance system^24^, that were both developed and custom-built in a collaborative project at the Rostock University Medical Center and the Karlsruhe Institute of Technology, respectively.

The CCM imaging is based on a Heidelberg Retina Tomograph (HRT III, Heidelberg Engineering, Heidelberg, Germany) as a confocal scanning laser unit. The original Rostock Cornea Module (RCM, Heidelberg Engineering, Heidelberg, Germany) is replaced by the alternative objective system RCM 2.0. Its software-controllable piezo drive can shift the focus plane within a total range of 500 µm and with a velocity of up to 800 µm/s. The RCM 2.0 is used to realize an automated continuous focus plane oscillation with a constant focus shift speed of 120 µm/s and an oscillation amplitude of ± 20 µm centered on the (manually chosen) initial position at the SNP level.

**Figure 1:**
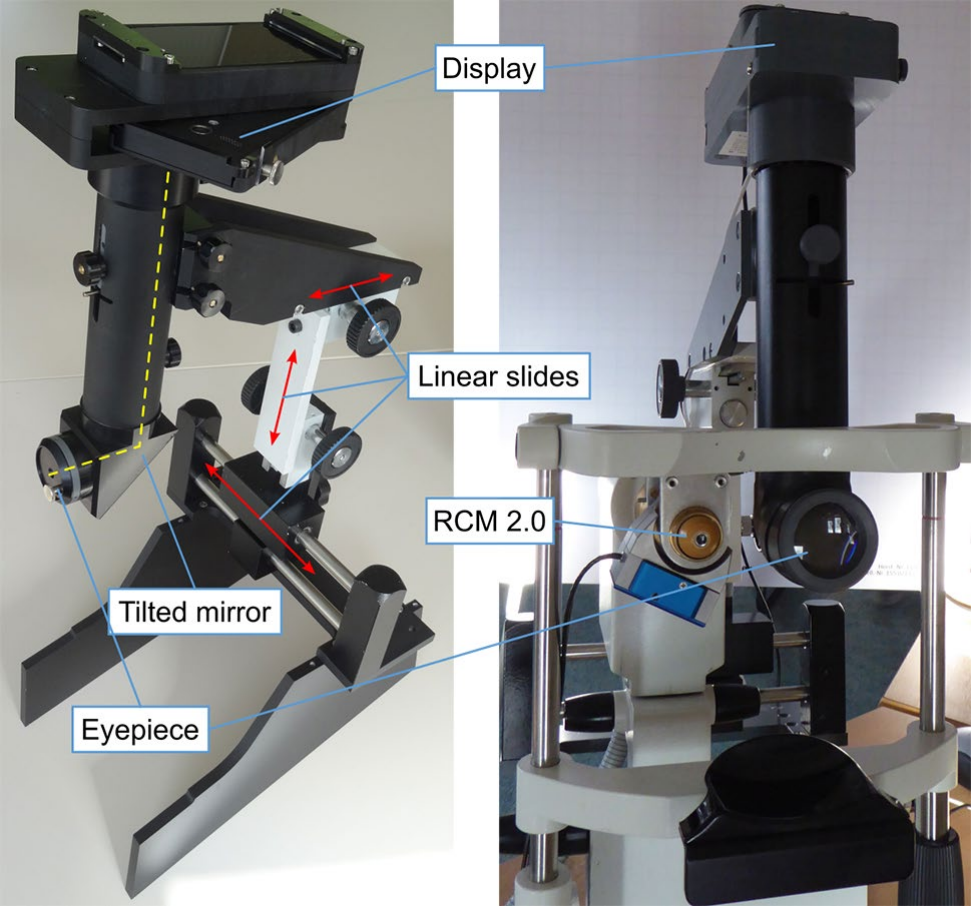
(Left) The EyeGuidance system. Dashed yellow line: optical axis. Red doublesided arrows: linear slides for individual positioning. (Right) The EyeGuidance system, mounted to an HRT confocal microscope. The HRT is equipped with an RCM 2.0.

The EyeGuidance system (cf. Fig. 1) shows a moving fixation target to the non-examined eye to guide the eye movements (of both eyes) along a continuously expanding spiral pattern. The tissue area covered by the CCM image sequence thus progresses similarly, expanding outwards from a central start point. In order to facilitate a reliable image registration process, the spiral parameters are carefully chosen such that several consecutive images along the spiral trajectory, as well as image pairs across adjacent spiral windings, overlap sufficiently^19^.

The automated imaging process with the RCM 2.0 and the EyeGuidance system requires specially adapted HRT software^20^. The two major changes with respect to the original software version are the possibility to record and export continuous CCM image sequences of arbitrary length with 30 fps and the addition of a network-based communication interface that facilitates the exchange of control signals (e.g. Start and Stop) and the live image data between the HRT software and our own software.

**Figure 2:**
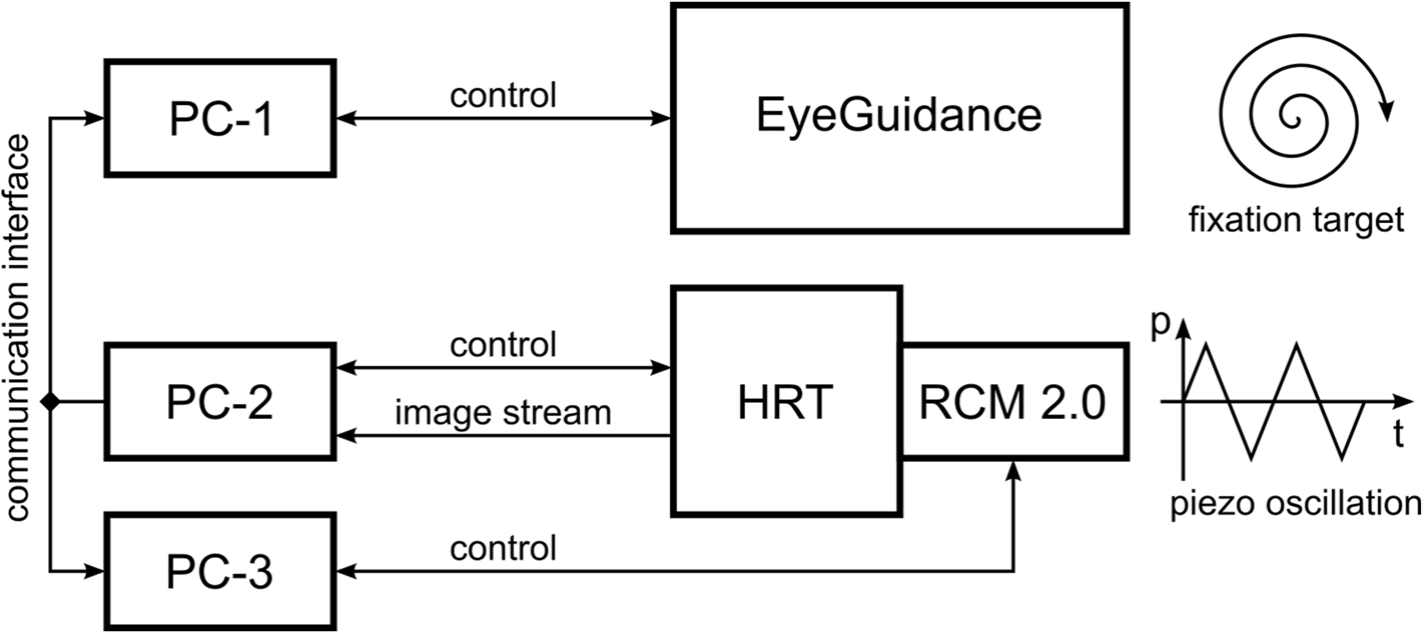
Schematic illustration of the CCM setup with focus plane control and guided eye movements (modified from^20^).

Figure 2 shows a schematic illustration of the entire setup for real-time large-area imaging of the SNP. Three dedicated PCs, connected via a TCP/IP network, are used to control the HRT (PC-2), the RCM 2.0 (PC-3) and the EyeGuidance system (PC-1). PC-1 also runs the online SNP mosaicking and tissue classification algorithms^25^. The three concurrent online tasks on PC-1, i.e. the EyeGuidance control process, the mosaicking module and the tissue classification module, run in separate operating system processes that also communicate over a TCP/IP interface. Microsoft Windows is used as the operating system on all three PCs. To realize real-time imaging, PC-1 is equipped with an AMD Ryzen 9 3950X CPU and 32 GB RAM. In addition to the real-time scenario, PC-1 can further be used to calculate large-area SNP montages offline from recorded datasets.

**Figure 3:**
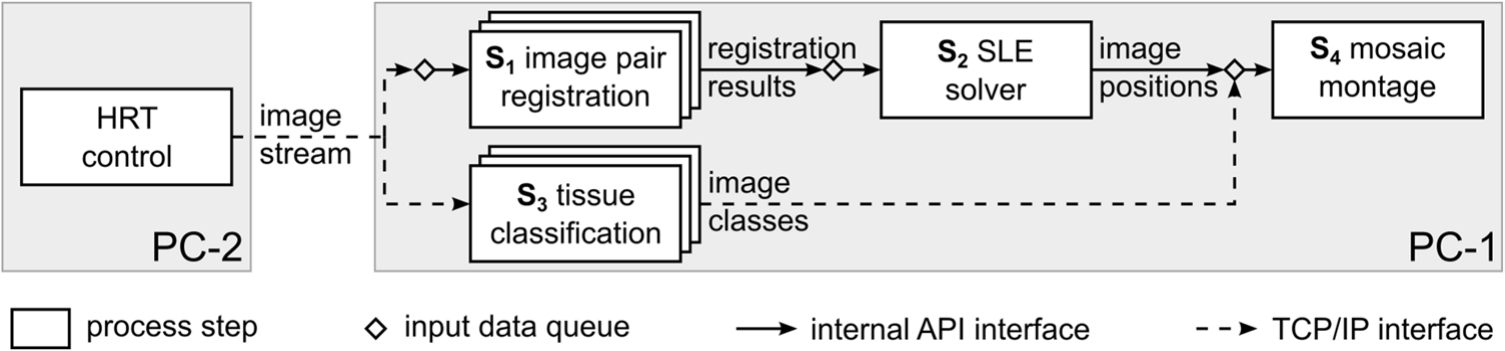
Image processing workflow for online mosaicking. The workflow consists of four separate functional modules that all work concurrently once data is passed through the processing pipeline. In addition, multiple concurrent instances of the modules for process steps S_1_ and S_3_ can process multiple data items at the same time. (SLE: system of linear equations).

The novel online mosaicking process is implemented in separate functional modules (cf. Fig. 3): image pair registration (step ${S}_{1}$), solving the system of equations (step ${S}_{2}$), tissue classification (step ${S}_{3}$) and montaging the mosaic image (step ${S}_{4}$). A description of the modules follows in “Image pair registration” through “Mosaic image montage”. The processing pipeline is implemented as an asynchronous workflow with all process steps of the pipeline running concurrently. When an intermediate process step ${S}_{i}$ finishes its task, the result data is stored in an input data queue of the subsequent step ${S}_{j}$ so ${S}_{i}$ can immediately continue processing the next piece of data in its own input queue.

### Image pair registration

For best result image quality, the offline mosaicking algorithm implements an exhaustive image registration approach that processes all possible combinations of two CCM images in a dataset^20^. This approach is characterized by a quadratic runtime complexity with respect to the dataset size; the precise number of image pair registrations is given by $\frac{N\left(N-1\right)}{2}$ for a dataset size of $N$ images. In the context of an online procedure, this means that the runtime required for each new image would increase linearly over time, which is not feasible in a real-time process considering that image pair registration is a relatively complex processing task. Effectively, the runtime available for registering a new image—i.e. the number of combinations that pair the new image with a previous image—needs to be limited by a constant threshold. For example, the computational resources of the used PC setup suffice for approximately 10 image pair registrations for each new image in a real-time application context; this number is of course specific to the used PC hardware.

Thus, a tailored approach for image pair selection is required for online mosaicking to replace the exhaustive approach. One overall aim is to use the available resources efficiently, i.e. to keep the number of unsuccessful image pair registrations low. A second overall aim is to establish successful registrations between overlapping images on adjacent spiral windings in order to support a high-quality mosaic image^19^.

The set of image pair registrations to be attempted for a given new image is determined by a combination of the following basic selection strategies that were developed along the motivations stated above. Despite the real-time constraints, it is beneficial for the robustness of the overall registration scheme and for an accurate motion correction to consider all images of the continuous image data stream for image pair registrations and to not exclude images based on the tissue classification results (“Tissue classification”).

#### Window strategy

**Figure 4:**
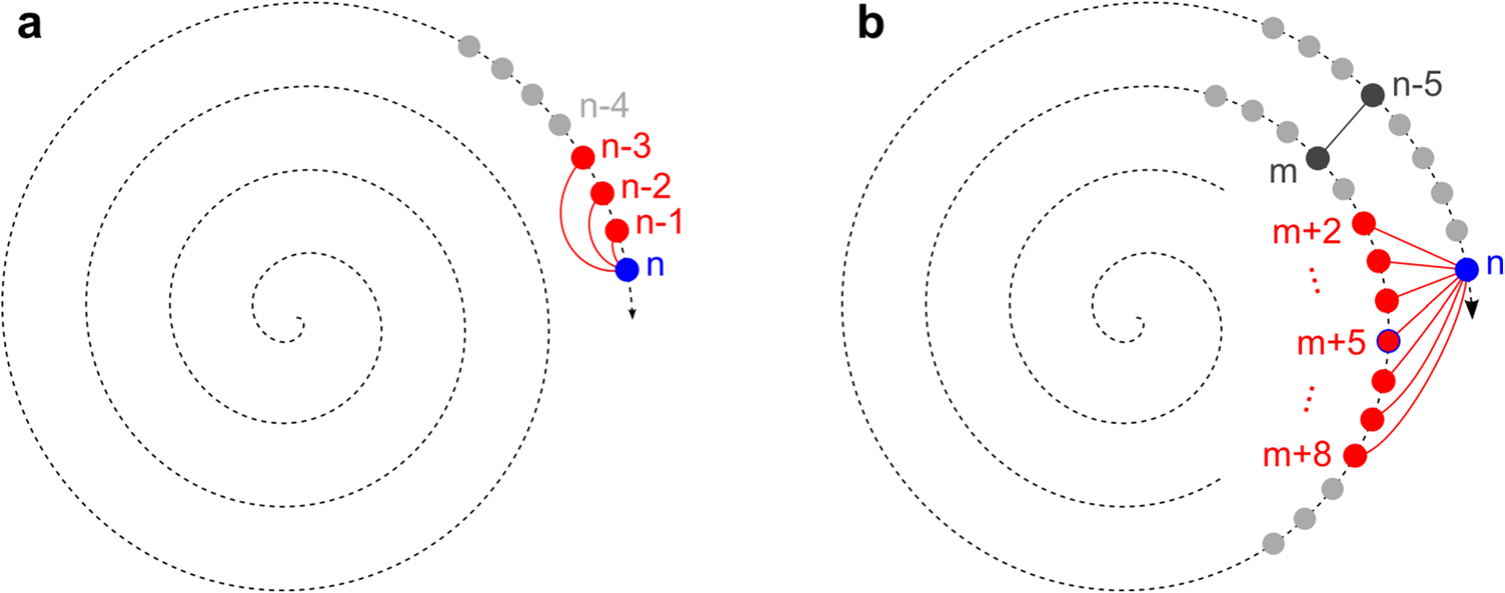
Strategies for image pair selection for the image registration module. In all examples, the blue dot represents a new acquired image $n$, the red dots represent previous images of the dataset that are paired with image $n$. (The distance between successive images is much smaller in reality than depicted in this figure.) **(a)** Window strategy: image $n$ is paired with the ${p}_{ww}$ preceding images (with ${p}_{ww}=3$). **(b)** Cross-winding strategy: If $\left(n-5,m\right)$ is the most recent successful cross-winding registration, then image $n$ is paired with image $m+5$ as well as the ${p}_{cw}$ images immediately preceding it and the ${p}_{cw}$ images immediately succeeding it (with ${p}_{cw}=3$).

The most fundamental selection strategy is called the “window strategy”. It pairs a new image with each of the immediately preceding images in the image sequence (see Fig. 4a), as these are assumed to possess the largest overlap with the new image. The number of image pairs to be selected in this way can be configured by the window width parameter ${p}_{ww}$. A single successful image pair registration is sufficient to establish a relation between the new image and the preceding images (and potentially the current mosaic image). The primary reason for registration failure here are motion artifacts in the image data induced by fast microsaccades^26^. If the registration for image pair $(n,n-1)$ is unsuccessful because image $n-1$ is affected by a microsaccade, a successful registration of image pair $(n,n-2)$ or $(n,n-3)$ can still link image $n$ to the preceding images. A single microsaccade is commonly limited to one image; rarely, two consecutive images are affected. By default, a value of ${p}_{ww}=3$ is therefore chosen to be able to effectively compensate microsaccades. This default value is used for Fig. 4a as well as for the experiments presented in “Results”. The window strategy is always active.

#### Cross-winding strategy

Small alignment errors from image pair registrations can add up over long registration chains. Thus, relying solely on image pair registrations along the spiral trajectory (window strategy) would lead to large misalignments between overlapping images on adjacent spiral windings. The registration of image pairs across adjacent spiral windings is therefore essential for a good mosaic image quality^19^. To select image pairs that have a high probability of a successful registration, we developed the so-called “cross-winding strategy”. Given a new image $n$, the first step is to search the immediately preceding images for successful cross-winding registrations (see Fig. 4b). In the example shown in Fig. 4b, the most recent successful cross-registration is found for the image pair $\left(n-5,m\right)$; image $m$ lies on the adjacent spiral winding. Based on this information, it can be hypothesized that the images $n$ and $m+5$ possess a large overlapping area. Because of the concurrent focus oscillation, however, it cannot be guaranteed that these two images are recorded at the same focus depth. If the focus depth differs too much, no valid registration can be achieved despite a large lateral overlap. Therefore, to consider the focus oscillation, image $n$ is not only paired with the calculated optimal neighbor image on the adjacent spiral winding, but also with the images immediately preceding and following it, which were recorded at different focus depths. The number of preceding and following images, respectively, can be specified by the cross width parameter ${p}_{cw}$. The default value is ${p}_{cw}=3$, as illustrated in Fig. 4b. Note that the value needs to be chosen with regard to the focus oscillation parameters in a way that results in a high probability that one of the $2{p}_{cw}+1$ selected cross-winding images originates from a similar focus depth as the new image $n$. For the focus oscillation parameters specified at the beginning of “Methods”, the $2{p}_{cw}+1=7$ images represent approximately one third of an oscillation period. This is enough to yield a reasonable probability for successful registration for at least one of the paired images, especially when considering that successful registrations are possible even with a focus depth difference of an image pair of up to approximately 8 µm due to the depth of field. The cross-winding strategy is the default case and always active whenever none of the following special case strategies is being used.

#### Anchor strategy

At the initial stage of the image recording, the cross-winding strategy is not suitable, because there is no adjacent spiral winding at first, and further because the curvature of adjacent spiral windings differs significantly for the innermost spiral part. However, most images still overlap with the first few images during this stage. The initial “anchor strategy” therefore pairs new images with the first ${p}_{aw}$ images, where ${p}_{aw}$ specifies the anchor width. For the same reason as with the window width parameter, a default value of ${p}_{aw}=3$ is commonly used. The anchor strategy is only active (in combination with the window strategy) throughout the initial phase up to an image index of ${p}_{init}$ (that defaults to ${p}_{init}=80$).

#### Sampling strategy

If there are still runtime resources available during the initial imaging phase, additional image pair registrations are attempted to relate the position of a new image $n$ with previous images evenly spaced throughout the entire dataset. The number of images to be registered in this “sampling strategy” is specified by the sample size ${p}_{ss}$, which needs to be chosen with regard to the used hardware. For instance, the window strategy and the anchor strategy only use up to 6 image pair registrations per new image, if the default parameters are used. If the limit of registrations per image is 10, a sample size of ${p}_{ss}=4$ could be used. In fact, the subsequent image processing steps require less runtime resources during the initial imaging phase, so that we use a value of ${p}_{ss}=8$ on our hardware. The sampling strategy is active whenever the anchor strategy is also used.

### Solving the system of equations

The result of each (successful) image pair registration provides information on the relative alignment between the respective image pair. More precisely, the registration result comprises relative translation vectors (i.e. position differences) between twelve horizontal sub-image slices of each of the images, in order to be able to describe motion-induced distortion artifacts in the images sufficiently well^26^. As a side note, we restrict the alignment analysis of image pairs to translations; even though the human eyes are anatomically able to rotate around their optical axis, we have not observed rotational components (in the image plane) in continuously recorded CCM datasets. The position differences constitute a system of (linear) difference equations that can finally be solved for the absolute sub-image positions in a global, common coordinate system, which is essentially the mosaic image coordinate system. The system matrix is always sparse—mainly because the successful registration results comprise only a small fraction of all image pair combinations, even in the case of the exhaustive image pair registration approach—and can be solved efficiently in a least squares sense, using an iterative CG (conjugated gradients) algorithm^27,28^.

This general procedure from the conventional, offline mosaic creation process is also used with two differences for the online case. First, the equation system has to be solved repeatedly throughout the imaging process in order to provide continuously updated image position information. As new registration results become available, they have to be introduced into the system of equations for the next execution cycle of the solver. Second, each cycle of the solver process must terminate within a limited duration (for the number of unknowns that are to be expected for reasonable dataset sizes) in order to not introduce a large amount of delay in the processing chain. This is achieved by limiting the solution accuracy adequately; to be specific, we limit the numerical accuracy to two decimal digits, i.e. a hundredth of a pixel. Note that it is also essential for runtime considerations to use the currently existing solution for the initial estimation of the next solver execution cycle.

### Tissue classification

The tissue classification software implements a Bag of Visual Words (BoVW) approach to calculate a feature vector for a given image, which is then classified by a set of support vector machines to distinguish between the cornea tissues (Epithelium, SNP, Stroma) as described in^25^. For the real-time setup with multiple modules on a single personal computer, the tissue classification software has been optimized for efficiency and runs as a standalone executable. It implements an asynchronous execution pattern, with one thread offering a network socket as a communication interface for the main mosaicking software, and a second thread that handles the image classification. Every image received over the network socket is forwarded to the classification or rejected if the classification is still in progress of processing a previous image. If required, multiple instances of the executable can run in parallel with the images being distributed alternately. Experiments have shown that two concurrent instances are able to process almost all images fast enough on the used hardware (rare cases of rejected images are probably attributable to the operating system process scheduler and can generally not be avoided reliably without a dedicated real-time operating system).

### Mosaic image montage

The calculated global image positions are finally used to compose a mosaic image from the single images. Only images classified as SNP (cf. “Tissue classification”) are included in the mosaic image, all others are excluded in this step. The general procedure is to calculate a fully aligned version of each SNP image, in which the motion artifacts are corrected and with the image at the correct location with respect to the mosaic image coordinate system. The fully aligned images ${I}_{n}$ are then montaged by weighted averaging

1 $$M=\frac{{M}_{n}}{{M}_{d}}=\frac{{\sum }_{n}{I}_{n}{w}_{n}}{{\sum }_{n}{w}_{n}}, \left({w}_{n}>0\right)$$

where ${w}_{n}$ is the weight function associated with ${I}_{n}$ and $M$ is the mosaicking result. The weight function is introduced to favor information from the high-contrast central image regions over information from the image borders, which usually exhibit a much lower signal-to-noise ratio.

As the mosaic image composition for a complete dataset, as in the offline implementation, can take up to several seconds, the real-time implementation of this process step requires a different approach. Instead of recalculating the entire mosaic image every time new information is available (i.e. for each new solution provided by the solver, cf. “Solving the system of equations”), only those regions affected by significant alterations are selectively updated. Such a change can be either the inclusion of a new image into the montage, or a significant change of the position information of an image that is already part of the montage. For this purpose, the mosaic image montage module implements two separate sub-modules that run concurrently. The first one continuously examines the solution updates for significant alterations (> 0.1 pixels) and sorts them in a priority queue. The inclusion of new images is always prioritized highest, position changes of present images are sorted by the absolute difference of their updated position with respect to their position in the current mosaic image visualization. The second sub-module continuously takes the highest-prioritized update item and processes it as follows.

The relevant data of each update item is the new position information of the sub-images of the given image (cf. “Solving the system of equations”), potentially complemented by the respective present values in the current mosaic image visualization. First, this information is expanded by spline interpolation to obtain the position coordinates, with respect to the mosaic image coordinate system, for each single image row of the image. The image rows are then included in the mosaic image. The weighted averaging mentioned above is implemented by keeping the numerator and denominator of Eq. (1) as separate intermediate images ${M}_{n}$ and ${M}_{d}$, respectively. The inclusion of a new image is then simply the addition of the respective data to both ${M}_{n}$ and ${M}_{d}$ for each single image row (rounding their position coordinates to the closest integer values) and to perform the division for only the affected mosaic image pixels. In case of a position update of an already included image, its previous contributions to the mosaic image have to be removed by subtracting the respective information from the intermediate images before the division.

## Results

The described real-time procedure is being used in several ongoing examination series^29^. Approval of the Ethics Committee of the University of Rostock in accordance with applicable laws, rules, and regulations was obtained on March 3, 2020 (A 2018-0162 with the amendment dated March 19, 2020). All performed examinations and experiments were in accordance with the applicable laws, rules, and regulations of the University of Rostock. Informed consent was obtained from all participants.

To date, 111 datasets of 15 different subjects have been collected with a typical image recording time of approx. 40 s (corresponding to approx. 1200 images). For these investigations, the registration strategies have always been parameterized by the default values given in “Image pair registration”. Several examples of the visual output of the real-time process are provided as Supplementary Videos S1 through S4.

**Figure 5:**
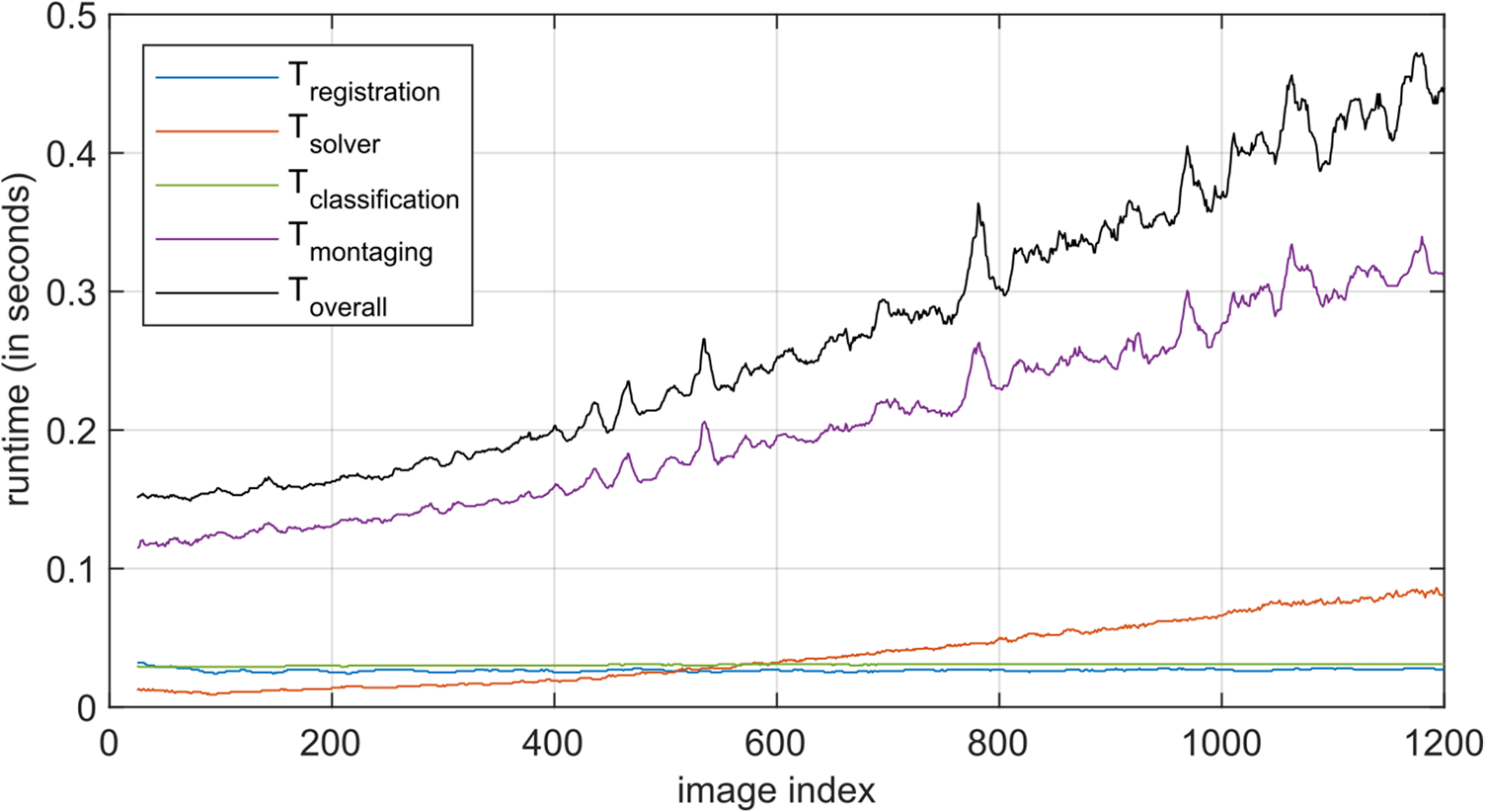
Processing time of the functional modules described in “Methods” with respect to the image index (median over datasets). The processing time of the image registration and tissue classification steps is independent of the image index. The processing time of the equation solver and mosaic montaging steps increases over the course of the imaging process.

Detailed runtime analyses were performed to assess and evaluate the real-time capability of the proposed method. The examination of the runtime behavior throughout the imaging process is of particularly interest with regard to the equation solver (step ${S}_{2}$) and the image montaging (step ${S}_{4}$) modules, as these exhibit increasing processing times per new image over the course of an examination. In contrast, the processing time per new image of the image registration (step ${S}_{1}$) and tissue classification (step ${S}_{3}$) modules is expected to remain constant. Figure 5 shows the processing times of the four functional modules with respect to the image index, averaged over the analyzed datasets.

For 10 image pair registrations per new image (with 4 registrations running concurrently in separate threads), the image registration step of the process pipeline requires an average of 27.0 ms (median) per image. The processing time for the tissue classification of an image was 30.0 ms. The runtime measurements for the online solver module determined an average duration per image of 13.0 ms at the beginning of the imaging process (averaged over 11 image indexes). The duration per image increased to 83.0 ms after 40 s (averaged over 11 image indexes). Finally, the required processing time of the montaging module increased from 120.5 ms to 313.1 ms (again averaged over 11 image indexes).

The average total time for an image to pass through the entire process chain, i.e. from becoming available on PC-1 after having been transferred from PC-2 (cf. Fig. 2) until its first appearance in the visualization of the online mosaic image, increases from 154.0 ms at the beginning to 444.1 ms after 40 s. Figure 5 shows that this total time is clearly dominated by the mosaic montaging process.

**Figure 6:**
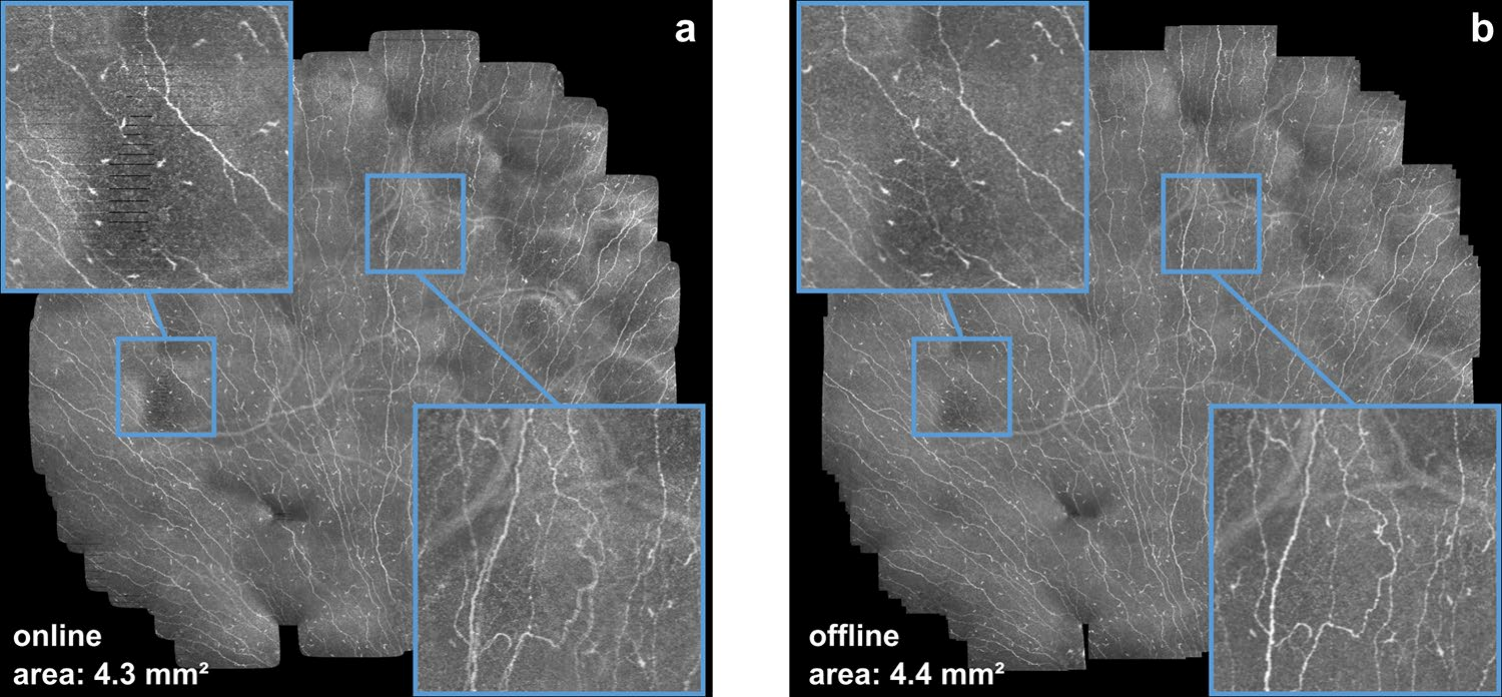
Comparison of the mosaic images (**(a)** online, **(b)** offline). The insets illustrate typical artifacts of the online mosaicking process and the improved image quality available through the additional offline process (The presented example is the third-largest mosaic image of the 111 collected datasets).

In order to evaluate the image quality of the online mosaicking process, the final mosaic images were compared with the results of the established offline process. Visual inspection shows that the cellular image features, particularly the nerve fibers, are clearly visible in the online image and that their global alignment is visually identical as in the offline image in general (see Fig. 6 and Supplementary Figs. S1 through S4). The visual comparison of image details (as in the insets in Fig. 6) reveals deficits of the online mosaic image, which are attributable to the smaller number of image pair registrations, the reduced precision of the solver and the occasional rejection of an image by the tissue classification module in the real-time process.

The area of the SNP region represented in the (final) online mosaic images was 2.20 ± 1.14 mm^2^ over all analyzed datasets. The corresponding offline mosaic images show a mean SNP area of 2.80 ± 0.89 mm^2^, the median ratio between final online and offline mosaic area is at 87.7% (minimum: 5.9%; lower quartile: 65.9%; upper quartile: 96.6%; maximum: 99.6%). The relative reduction of the visible SNP area in the online process is primarily attributable to the limited amount of image pair registrations.

## Conclusion

Several methods for large-area imaging of the SNP have been published over the course of the last ten years, some of them proposing automated workflows for the imaging process^16,17,19,20^. One publication described a real-time SNP mapping procedure that required manual interaction with the microscope^15^. To the best of our knowledge, the present contribution is the first to combine both aspects in an integrated system that supports a fully automated image acquisition process with a real-time montage of the captured SNP region.

The manual interaction with the presented system is limited to positioning the field of view at an appropriate location of the SNP. After that, the imaging process itself is completely automated. Aside from the the initial manual positioning step, the operator’s level of experience with the system has no influence on the resulting image data.

The current system setup as described, consisting of three separate PCs controlling the HRT, the RCM 2.0 and the EyeGuidance system (cf. Fig. 2), imposes significant space constraints, which can be tolerated in an experimental research environment, but limit the general applicability in clinical practice. More work towards system integration will be necessary to reduce the space requirements as well as the technical effort required to install and start the overall system.

Given that the time required by an image to pass through the entire process pipeline—its delay—increases continuously over the imaging duration, the term “real-time” may not apply strictly to the described process. We argue that the term is still applicable in a less strict sense, as the delay in the order of 500 ms at the final stages of a 40 s imaging period was hardly noticed by the operator in the experiments. However, this assessment may have to be revised for scenarios with significantly longer imaging duration.

The presented method has been tested successfully in more than 110 examinations of 15 volunteers and selected patients with recording times of approximately 40 s. The online mosaic image provides a good overview of the captured SNP region. Its primary purpose is to offer the operator the necessary information to evaluate the quality of the recorded image dataset immediately during the recording process. This allows for the first time an informed decision of whether or not the recorded dataset is sufficient or the examination needs to be repeated. In cases of insufficient data quality, the operator can also quickly abort and repeat an examination immediately, while the patient is still at the microscope. This helps reduce the required examination times for the patients.

In addition to the evaluation of the mosaic image size and quality, online mosaicking also offers the chance to identify an overlap of the imaged tissue region with previous SNP mosaic images immediately at the time of recording; this has proven to be particularly advantageous for follow-up examinations^29^.

The online mosaic image quality may often be sufficient for a first, ad hoc qualitative assessments of the SNP by the examining clinician, despite small misalignment artifacts, such as in the bottom right inset in Fig. 6a. In some cases, misalignment artifacts affect larger portions of the image (cf. Supplementary Fig. S2 and Supplementary Video S2), which may impair an ad hoc qualitative assessment of the SNP condition. The misalignment artifacts are directly related to the limited number of image pair registrations, i.e. some specific image pairs that would solve the misalignment are not selected for registration by the implemented image pair selection strategies. One potential way to reduce the misalignment artifacts would therefore be to develop and implement optimized selection strategies which are able to identify specifically such image pairs that greatly improve the online image quality (without increasing the total number of registered image pairs). Beyond a first qualitative overview of the SNP, however, reliable qualitative assessments and subsequent quantitative analyses—and image processing algorithms in particular—should generally use the best possible data available. The routine practice therefore is to calculate the offline SNP montage for at least one recorded dataset per examination, in order to benefit from the superior image quality. In fact, the processing time for the offline montage of about 2–3 min for a dataset of 1200 images is not a serious limitation in most scenarios.

Although the presented system is predominantly used for SNP mosaicking, as described herein, it can also be employed in a depth scan mode with larger focus scan depth (up to a maximum of 500 µm) and reduced lateral extent. For this operation mode, the tissue classification module is deactivated and the mosaic montage module is replaced by a module that uses the same basic algorithms but creates $x$–$z$- and $y$–$z$-montages in addition to a $x$–$y$-mosaic. The main difference is that—by adding a $z$-coordinate to each recorded image—the motion-corrected image data is mapped onto a 3D voxel grid instead of the 2D pixel grid. The visualized real-time $x$–$z$- and $y$–$z$-montages are sections through that volume. The depth scan mode is presently being evaluated for imaging extended sections of the epithelium, including the documentation and quantification of the complex morphology of corneal epithelial structures. These cell layers with a turnover time of only a few days are highly dependent on the status of the SNP, and its understanding will most likely substantially help understand the pathophysiology of ocular surface diseases^30^. As with the online SNP mosaic images, the real-time depth sections provide an adequate basis for the operator to assess the size and quality of the acquired dataset at the time of the recording. Precise offline 3D-registration techniques have to be developed to generate high-quality volume image data of the corneal tissue.

## Supplementary Information

Supplementary Figures.

Supplementary Video 1.

Supplementary Video 2.

Supplementary Video 3.

Supplementary Video 4.

Supplementary Legends.

## Data Availability

The data presented in this study are available on reasonable request from the corresponding author.
