# Supplementary material for: Real-time large-area imaging of the corneal subbasal nerve plexus
Source: Sci Rep. 2022 Feb 15;12:2481. doi: 10.1038/s41598-022-05983-5 (PMC8847362; doi:10.1038/s41598-022-05983-5)
Supplement: Supplementary file 1 — Supplementary Figures. [file 41598_2022_5983_MOESM1_ESM.pdf]

Real-time large-area imaging of the corneal subbasal nerve plexus

Stephan Allgeier*[1], Andreas Bartschat[1], Sebastian Bohn[2,3], Rudof F. Guthoff[2], Veit Hagenmeyer[1], Lukas Kornelius[1], Ralf Mikut[1], Klaus-Martin Reichert[1], Karsten Sperlich[2,3], Nadine Stache[2,4], Oliver Stachs[2,3], Bernd Köhler[1]

[1] Institute for Automation and Applied Informatics, Karlsruhe Institute of Technology (KIT), Karlsruhe, Germany

[2] Department of Ophthalmology, Rostock University Medical Center, Rostock, Germany

[3] Department Life, Light & Matter, University of Rostock, Rostock, Germany

[4] Department of Obstetrics and Gynecology, University of Rostock, Rostock, Germany

*Corresponding author:

Stephan Allgeier
Karlsruhe Institute of Technology (KIT)
Institute for Automation and Applied Informatics
Hermann-von-Helmholtz-Platz 1
D-76344 Eggenstein-Leopoldshafen
Germany

Phone: +49-721-608-23172

e-mail: stephan.allgeier@kit.edu

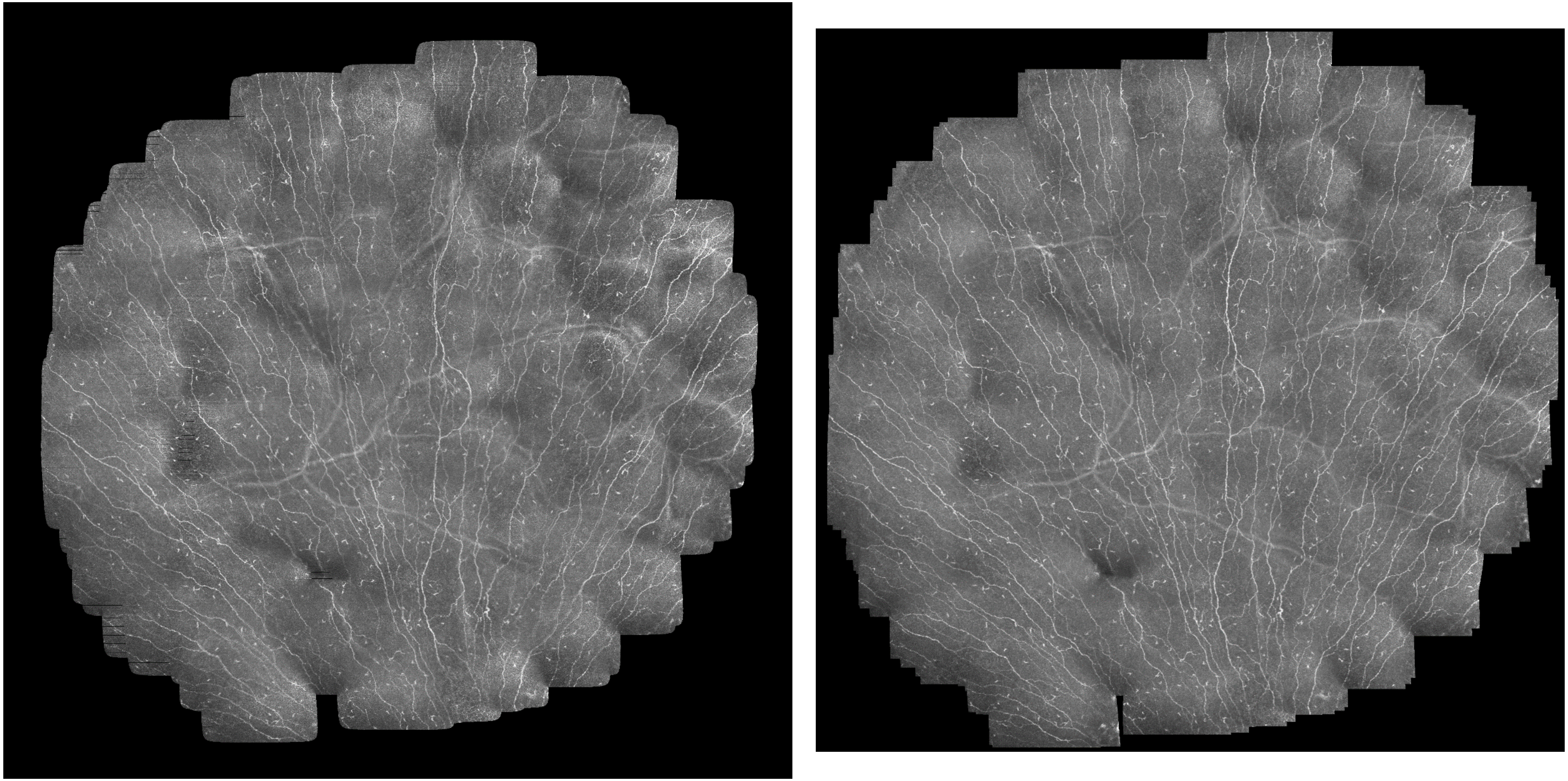

Supplementary Figure S1: Mosaic images generated from a dataset; example 1; (left) final mosaic image from the online process; (right) mosaic image from the offline process. (See Supplementary Video S1 for the accompanying online process video.)

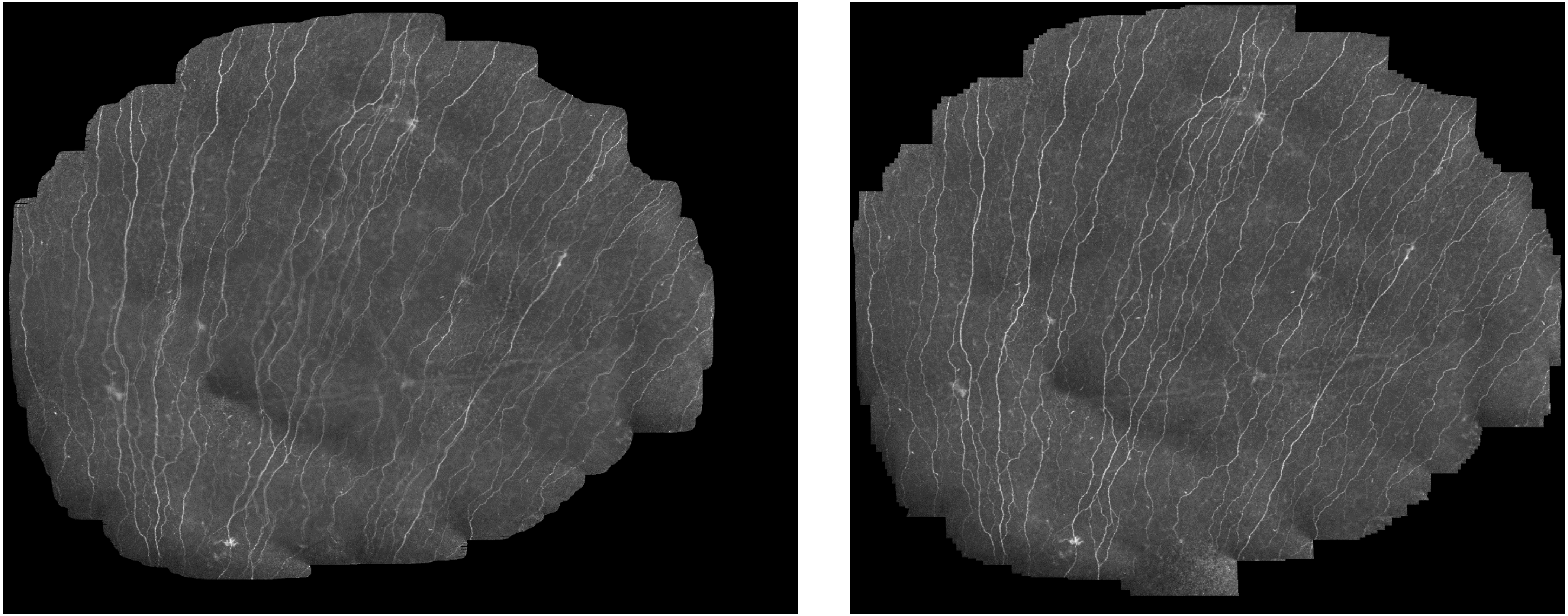

Supplementary Figure S2: Mosaic images generated from a dataset; example 2; (left) final mosaic image from the online process; (right) mosaic image from the offline process. (See Supplementary Video S2 for the accompanying online process video.)

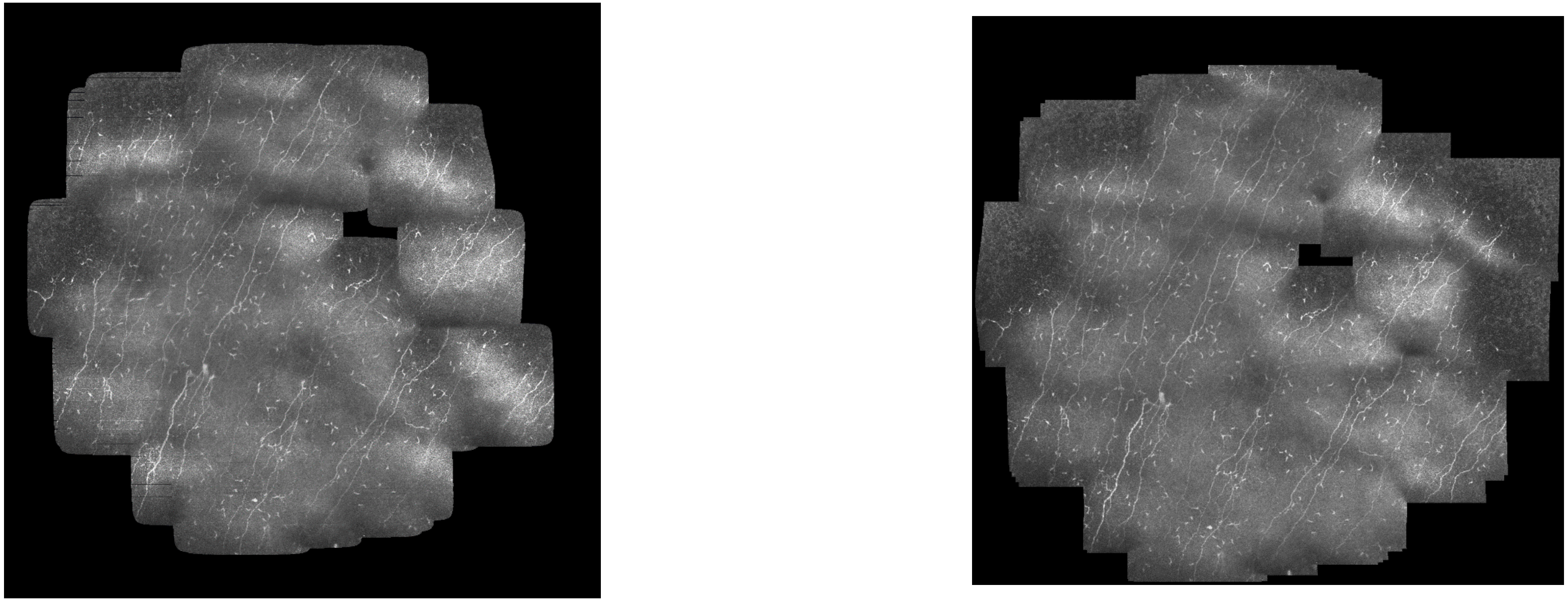

Supplementary Figure S3: Mosaic images generated from a dataset; example 3; (left) final mosaic image from the online process; (right) mosaic image from the offline process. (See Supplementary Video S3 for the accompanying online process video.)

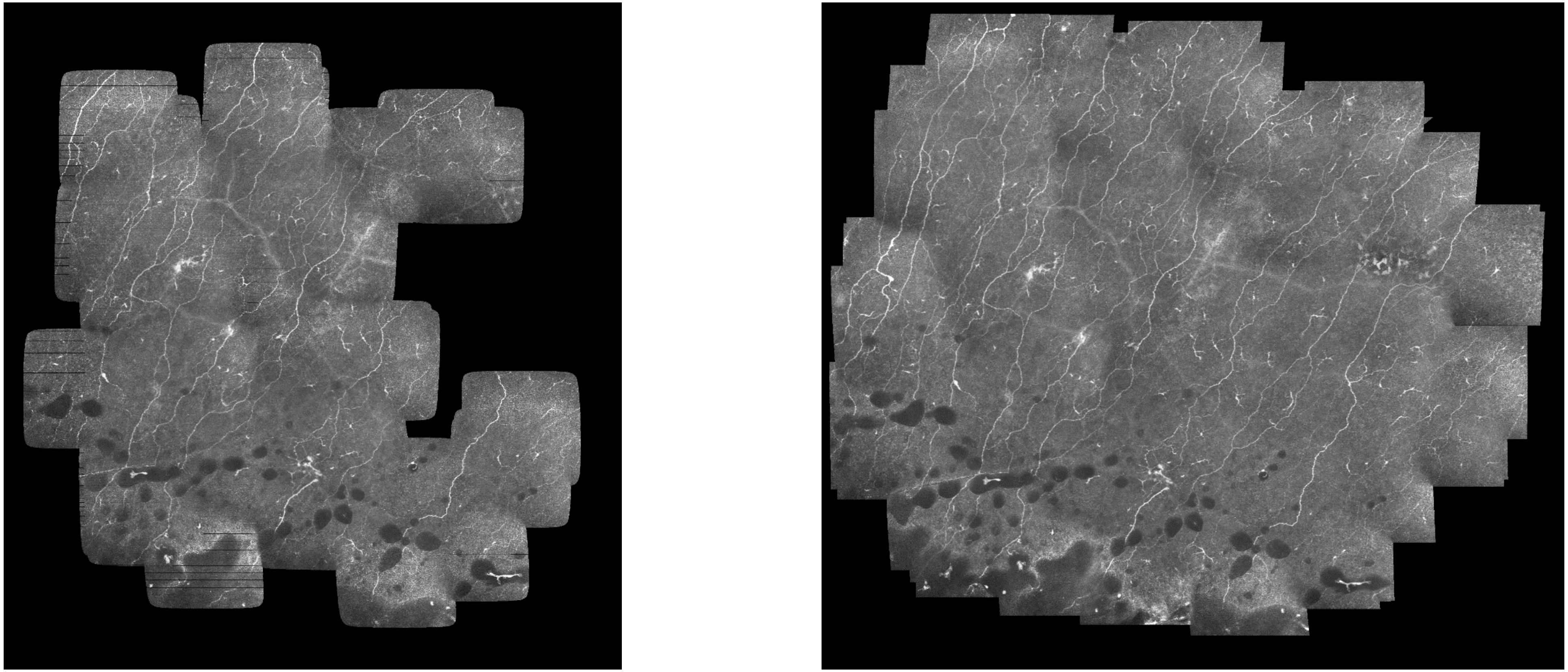

Supplementary Figure S4: Mosaic images generated from a dataset; example 4; (left) final mosaic image from the online process; (right) mosaic image from the offline process. (See Supplementary Video S4 for the accompanying online process video.)
