# Supplementary material for: Real-time large-area imaging of the corneal subbasal nerve plexus
Source: Sci Rep. 2022 Feb 15;12:2481. doi: 10.1038/s41598-022-05983-5 (PMC8847362; doi:10.1038/s41598-022-05983-5)
Supplement: Supplementary file 6 — Supplementary Legends. [file 41598_2022_5983_MOESM6_ESM.pdf]

Real-time large-area imaging of the corneal subbasal nerve plexus

Stephan Allgeier*[1], Andreas Bartschat[1], Sebastian Bohn[2,3], Rudof F. Guthoff[2], Veit Hagenmeyer[1], Lukas Kornelius[1], Ralf Mikut[1], Klaus-Martin Reichert[1], Karsten Sperlich[2,3], Nadine Stache[2,4], Oliver Stachs[2,3], Bernd Köhler[1]

[1] Institute for Automation and Applied Informatics, Karlsruhe Institute of Technology (KIT), Karlsruhe, Germany

[2] Department of Ophthalmology, Rostock University Medical Center, Rostock, Germany

[3] Department Life, Light & Matter, University of Rostock, Rostock, Germany

[4] Department of Obstetrics and Gynecology, University of Rostock, Rostock, Germany

*Corresponding author:

Stephan Allgeier
Karlsruhe Institute of Technology (KIT)
Institute for Automation and Applied Informatics
Hermann-von-Helmholtz-Platz 1
D-76344 Eggenstein-Leopoldshafen
Germany

Phone: +49-721-608-23172

e-mail: stephan.allgeier@kit.edu

Supplementary Video S1: Screencast video of the online mosaicking process; example dataset 1. (See Supplementary Figure S1 for a side-by-side comparison of the final online mosaic image and the corresponding offline mosaic image.)

Supplementary Video S2: Screencast video of the online mosaicking process; example dataset 2. (See Supplementary Figure S2 for a side-by-side comparison of the final online mosaic image and the corresponding offline mosaic image.)

Supplementary Video S3: Screencast video of the online mosaicking process; example dataset 3. (See Supplementary Figure S3 for a side-by-side comparison of the final online mosaic image and the corresponding offline mosaic image.)

Supplementary Video S4: Screencast video of the online mosaicking process; example dataset 4. (See Supplementary Figure S4 for a side-by-side comparison of the final online mosaic image and the corresponding offline mosaic image.)
